# Transcriptional abnormalities in induced pluripotent stem cell-derived oligodendrocytes of individuals with primary progressive multiple sclerosis

**DOI:** 10.3389/fncel.2022.972144

**Published:** 2022-09-28

**Authors:** Melanie J. Plastini, Haritha L. Desu, Maureen C. Ascona, Anna L. Lang, Mario A. Saporta, Roberta Brambilla

**Affiliations:** ^1^The Miami Project to Cure Paralysis, Department of Neurological Surgery, University of Miami Miller School of Medicine, Miami, FL, United States; ^2^The Neuroscience Graduate Program, University of Miami Miller School of Medicine, Miami, FL, United States; ^3^Department of Neurology, University of Miami Miller School of Medicine, Miami, FL, United States; ^4^Department of Neurobiology Research, Institute of Molecular Medicine, University of Southern Denmark, Odense, Denmark; ^5^BRIDGE-Brain Research-Inter-Disciplinary Guided Excellence, Department of Clinical Research, University of Southern Denmark, Odense, Denmark

**Keywords:** oligodendrocytes, human induced pluripotent stem cells, primary progressive multiple sclerosis, RNA-sequencing, inflammasome, NLRP2

## Abstract

Multiple sclerosis (MS) is the most common neurological disorder in young adults and is classically defined as a chronic inflammatory demyelinating disease of the central nervous system (CNS). Although MS affects millions of people worldwide, its underlying cause remains unknown making discovery of effective treatments challenging. Whether intrinsic or extrinsic factors contribute to MS initiation and progression is still unclear. This is especially true for primary progressive MS (PPMS), the rarest form of the disease, in which progressive and irreversible loss of neurological function is often observed in the absence of an overt immune-inflammatory response. To test the hypothesis that intrinsic dysfunction in oligodendrocytes (OLs), the primary targets of damage in MS, may contribute to PPMS etiopathology, we differentiated human induced pluripotent stem cell (hiPSC) lines derived from PPMS and healthy individuals into mature OLs to compare their transcriptional profile. PPMS derived OLs displayed hundreds of differentially expressed genes compared to control OLs, many associated with cell adhesion, apoptosis and inflammation, including the inflammasome component *Nlrp2*, which was highly upregulated. NLRP2 immunoreactivity in OLs was confirmed in post-mortem PPMS brain tissues, with higher expression than in control tissues. Altogether, our findings suggest that mature OLs in PPMS affected individuals carry intrinsic abnormalities that could contribute, at least in part, to the pathophysiology of this form of the disease.

## Introduction

Multiple sclerosis (MS) is a chronic demyelinating disease of the central nervous system (CNS) (Nylander and Hafler, 2012) and the most common non-traumatic neurological disorder in young adults, affecting an estimated 1 million people in the United States alone (Wallin, 2017; Reich et al., 2018; Wallin et al., 2019). Its cause remains elusive, although it is established that MS manifests in genetically susceptible individuals with environmental factors influencing penetrance and clinical course (Reich et al., 2018). This includes Epstein–Barr virus infection, which a recent longitudinal study determined not only to greatly increase the risk of subsequent MS but to precede the development of the disease, supporting a pathogenic role (Bjornevik et al., 2022). MS develops with distinct clinical phenotypes, the most frequent of which is relapsing remitting MS (RRMS, 85% of cases at onset), pathologically characterized by inflammatory demyelinating lesions in the white and gray matter, heavily infiltrated with immune cells (Frohman et al., 2006). In over 75% of cases, RRMS evolves into secondary progressive MS (SPMS), where individuals experience irreversible accumulation of disability associated with neurodegeneration (Nylander and Hafler, 2012). In a small percentage of cases, primary progressive multiple sclerosis (PPMS), develops where irreversible and progressive neurodegeneration starts at onset (Lassmann et al., 2012). In progressive MS forms, chronic demyelinated lesions with axonal loss accumulate in the white matter, but diffuse changes also occur in the seemingly unaffected white matter, commonly referred to as normal appearing white matter (NAWM). While major progress has been made in understanding disease mechanisms in RRMS, those driving progressive MS remain largely unresolved, which explains why effective treatment options are available for RRMS, but virtually none for PPMS or SPMS. This is a major unmet need.

Whether MS is initiated by a peripheral and/or CNS trigger is still unclear (Dendrou et al., 2015; Axisa and Hafler, 2016). According to the so-called “CNS extrinsic” hypothesis of MS, autoreactive T cells enter the CNS where they activate the innate immune response and cause demyelination. This is well suited in modeling RRMS (Trapp and Nave, 2008; Dendrou et al., 2015) but does not accurately fit the pathophysiology of PPMS and SPMS where axonal damage, demyelination, and neurodegeneration are often independent of immune cell presence. PPMS, especially, seems to better fit with the so-called “CNS intrinsic” model, where events initiated in the CNS are believed to trigger the disease (Trapp and Nave, 2008; Dendrou et al., 2015; Mahad et al., 2015). In line with this hypothesis, in PPMS oligodendrocyte (OL) death and demyelination have been shown to occur in the absence of T cells. In PPMS, hypoxia-like demyelinating lesions (pattern III), characterized by axonal transection and histopathological similarities to stroke-type tissue injury, are often found (Lucchinetti et al., 2000; Peterson et al., 2001; Aboul-Enein et al., 2003). Here, demyelination is initiated by degeneration of the most distal OL processes, leading to OL apoptosis. This evidence and more recent single-nucleus RNA sequencing (snRNA-seq) studies of post-mortem MS tissues (Falcao et al., 2018; Jäkel et al., 2019) suggest that primary OL dysfunction may play a role in PPMS etiopathogenesis.

To investigate this possibility, we took an *in vitro* approach using human induced pluripotent stem cell (hiPSC) lines derived from PPMS and healthy individuals and differentiated them into mature myelinating oligodendrocytes (OLs) to map and compare their transcriptional profile by RNA sequencing (RNA-seq). PPMS derived cells showed hundreds of differentially expressed genes compared to control cells, many associated with adhesion, apoptosis, and inflammation, including the inflammasome component *Nlrp2*, which was highly upregulated. Immunolabeling for NLRP2 in OLs was confirmed in post-mortem PPMS brains, with higher expression than in control brains. Altogether, our findings suggest that mature OLs in PPMS affected individuals carry intrinsic abnormalities that could contribute, at least in part, to the pathophysiology of this form of the disease.

## Materials and methods

### Post-mortem human brain tissues

Unfixed fresh-frozen brain tissues were obtained from the United Kingdom Multiple Sclerosis Tissue Bank at Imperial College London *via* a United Kingdom prospective donor scheme with full pre-mortem consent and ethical approval (United Kingdom Ethics Committee 08/MRE09/31). Normal white matter (NWM) areas from six non-neurological control cases and NAWM areas from six PPMS cases were analyzed. Demographical and clinical data for each tissue donor are provided in Table 1.

**TABLE 1:** Demographical and clinical data of control and primary progressive multiple sclerosis (PPMS) tissue donors.

Case #	Sex	Age	Disease duration (years)	Tissue type	Diagnosis
MS070	F	77	19	NAWM (brain)	PPMS
MS129	F	67	24	NAWM (brain)	PPMS
MS168	F	88	30	NAWM (brain)	PPMS
MS216	F	53	9	NAWM (brain)	PPMS
MS313	M	66	18	NAWM (brain)	PPMS
MS576	M	75	31	NAWM (brain)	PPMS
CO22	F	69	N/A	WM (brain)	Control (non-neurological)
CO28	F	60	N/A	WM (brain)	Control (non-neurological)
CO30	M	75	N/A	WM (brain)	Control (non-neurological)
CO36	M	68	N/A	WM (brain)	Control (non-neurological)
CO45	M	77	N/A	WM (brain)	Control (non-neurological)
CO54	M	66	N/A	WM (brain)	Control (non-neurological)

NAMW, normal appearing white matter; WM, white matter.

### Human induced pluripotent stem cell differentiation

Human induced pluripotent stem cell (hiPSC) lines from two PPMS and two control individuals were obtained from the New York Stem Cell Foundation and an additional control hiPSC line was kindly provided by Dr. Mario Saporta. All cell lines were extensively validated in previous studies (Douvaras et al., 2014; Douvaras and Fossati, 2015; Saporta et al., 2015). Control and PPMS lines were age, sex, and ethnicity matched to the best of our ability. Specific information on each line is reported in Table 2. Differentiation of hiPSCs into OLs was carried out according to the “fast protocol” published by Douvaras and Fossati (2015). Briefly, hiPSCs were thawed and expanded in StemFlex medium (ThermoFisher, Waltham, MA, United States) to form colonies for differentiation. Colonies were then dissociated into single cells using Accutase (Life Technologies, Carlsbad, CA, United States, #A11105-01), seeded in poly-L-ornithine- and laminin-coated 6-well plates at 1 × 10^5^ cells/well density, and cultured in StemFlex medium for 2 days before differentiation, which was induced by switching to neural induction medium (NIM) containing freshly added SB434542 (TGFβ inhibitor), LDN-193189 (BMP inhibitor), and retinoic acid (RA) (day 0). Medium was changed daily for 8 days. From day 8 to 12, cells were exposed to RA and smoothened agonist (SAG). On day 12, cells were mechanically dissociated, transferred to non-coated 6-well plates and grown in suspension until day 20. On day 20, cells were switched to PDGF containing medium until day 30. On day 30, cell aggregates were replated onto poly-L-ornithine- and laminin-coated 6-well plates, switched to mitogen-free medium, and grown adherent for the rest of the differentiation through day 75. On Day 76, cultures were immunolabeled for O4 and FACS-sorted either for RNA extraction and subsequent RNA-seq and qPCR, or to be replated onto 24-well plates containing poly-L-ornithine- and laminin-coated coverslips to perform immunostaining and high content analysis (HCA). For each hiPSC line, a minimum of four 6-well plates were obtained per differentiation. From these plates, a minimum of 4–6 wells/line/differentiation were used for FACS-sorting of the O4^+^ population. Cells were then pooled after sorting. For immunocytochemistry, cells were replated onto two 24-well plates on day 30 with 3–4 aggregates per well. One plate was labeled directly at day 76 to assess population distributions at the end of differentiation. The other plate was set up with poly-L-ornithine- and laminin-coated coverslips in each well for the cells to differentiate on. At day 76, once fixed and immunostained, coverslips were transferred to slides in order to examine cell morphology and expression of specific proteins at higher magnification.

**TABLE 2:** Human induced pluripotent stem cell (hiPSC) lines used for oligodendrocyte (OL) differentiation.

Cell line ID	Sex	Age at biopsy	Ethnicity	Diagnosis	Passage number
060104-01-MR (NYSCF)	F	62	White	PPMS	17
060107-01-MR (NYSCF)	M	61	White	PPMS	35
050592-01-MR (NYSCF)	M	65	White	Control	8
050659-01-MR (NYSCF)	F	65	White	Control	8
PIZ3.13 (MAS)	M	10	White	Control	18

### Cytological and histological analyses

#### Immunocytochemistry

Cells were fixed with 4% paraformaldehyde (PFA) in 0.1 M PBS and blocked with 5% normal goat serum in 0.1 M PBS with 0.1% Triton-X for 1 h at room temperature. Cells were then incubated overnight at 4°C with primary antibodies against Olig2 (Millipore, Burlington, MA, United States, #AB9610), MBP (Millipore, Burlington, MA, United States, #MAB386), Iba1 (FUJIFILM Wako Chemicals USA, Richmond, VA, United States, #019-19741), GFAP (Dako-Agilent, Santa Clara, CA, United States, #Z0334; Invitrogen, Waltham, MA, United States, #13-0300), and NeuN (Millipore, Burlington, MA, United States, #MAB377). For immunolabeling of O4^+^ OLs, live cells were incubated with the supernatant of a hybridoma cell line producing O4 monoclonal antibody kindly provided by Dr. Paula Monje (Peng et al., 2020) for 15 min at room temperature prior to fixation. Immunoreactivity was visualized with secondary species-specific IgG or IgM fluorescent antibodies in 0.1 M PBS with 0.1% Triton-X and 5% normal goat serum for 1 h at room temperature (1:1000; Alexa Fluor-488, Alexa Fluor-594, and Alexa Fluor-647, Invitrogen, Waltham, MA, United States). Nuclei were then stained with DAPI for 3 min at room temperature (1:1000 in 0.1 M PBS, Invitrogen, Waltham, MA, United States). Representative images were taken with a Zeiss Axiovert A1 fluorescence microscope or with a Leica TCS SP5 confocal microscope at 60x magnification for cells grown on coverslips. Quantification of cell percentages was performed in an unbiased manner from 9 randomly selected fields of view per technical replicate at 10x magnification using the Cellomics ArrayScan VTI HCA system (ThermoFisher, Waltham, MA, United States). At least 20,000–30,000 cells were analyzed per differentiation.

#### Immunohistochemistry

Fresh-frozen human brain tissue blocks from the United Kingdom Multiple Sclerosis Tissue Bank were serially sectioned across 10 slides at a 10 μm thickness. Two sections per slide were collected. O4 immunolabeling was conducted before fixation at room temperature for 15 min. Sections were blocked with 5% normal goat serum in 0.1 M PBS with 0.4% Triton-X for 1 h and incubated overnight at 4°C with primary antibodies against GFAP (Dako-Agilent, Santa Clara, CA, United States, #Z0334) and NLRP2 (NALP2, Invitrogen, Waltham, MA, United States, #PA5-29196). Staining was visualized with secondary species-specific fluorescent antibodies in 0.1 M PBS with 0.1% Triton-X and 5% normal goat serum for 1 h at room temperature (1:1000, goat anti-mouse Alexa Fluor 488 and 594, goat anti-rabbit Alexa Fluor 488 and 594, and goat anti-rat 488, 594, and 647, Invitrogen, Waltham, MA, United States). After staining nuclei with DAPI, sections were coverslipped and imaged with a Leica TCS SP5 confocal microscope at 40x magnification. At least three sections per sample were analyzed, from which representative images where chosen.

#### Hematoxylin and eosin staining

Human brain tissue was fixed for 15 min in cold 4% PFA. Sections were washed and stained with an hematoxylin and eosin (H&E) staining kit (Abcam, Cambridge, United Kingdom, #ab245880), according to manufacturer’s instructions. Briefly, sections were incubated in Hematoxylin solution for 3 min, then, after washes, in Bluing Reagent for 20 s, followed by ethanol washes and Eosin Y Solution for 30 s. Sections were then dehydrated and cleared with xylene (three changes, 1 min each), mounted with DPX mounting medium, coverslipped, and imaged with a PrimeHisto XE Histology Slide Scanner.

### Sholl analysis of cell complexity

Analysis was performed on O4^+^ cells grown on poly-L-ornithine- and laminin-coated coverslips until day 76 of differentiation. After immunostaining for O4 (see above), cells were fixed with 4% PFA, labeled with DAPI, and coverslips mounted onto glass slides for imaging. Images were randomly acquired from 3 replicates/group at 20x magnification with a Zeiss Axiovert fluorescence microscope and a minimum of 25 cells/group were selected for morphological analysis, making sure that cells were sufficiently distanced to avoid process overlap. Sholl analysis of cell complexity was performed by a blinded investigator using Image J software. After manual tracing of individual cells, the number of “nodes” or “process intersections” was automatically estimated at increasing distance from the cell center (4 μm increments). Total process length/cell was also quantified.

### Fluorescence activated cell sorting

Fluorescence activated cell sorting (FACS) was performed after differentiation. Adherent cultures were incubated with Accutase and gently dissociated. Cells were then spun down, resuspended in 50 μl DMEM/F12 + 0.5 μl TruStain FcX Fc Receptor Blocking Solution (BioLegend, San Diego, CA, United States), incubated at room temperature for 5 min, then immunolabeled with anti-O4-APC (Miltenyi, Gaithersburg, MD, United States, #130-119-155) for 45 min on ice. Live cells were then sorted with a MoFlo Astrios EQ cell sorter (Beckman-Coulter, Brea, CA, United States).

### Semi-quantitative real-time RT-PCR

Ribonucleic acid from FACS-sorted cells was isolated using the Arcturus PicoPure RNA Isolation Kit (Applied Biosystems, Waltham, MA, United States, #KIT0204) according to the manufacturer’s protocol. Sorted O4^+^ cells were resuspended in 100 μl RNA extraction buffer and incubated at 42°C for 30 min. Samples were spun down and the RNA-containing supernatant further purified of residual genomic DNA by on-column digestion with RNase-free DNase (Qiagen, Germantown, MD, United States, #79254). Total RNA (5 μg) was reverse transcribed with Sensiscript RT Kit (Qiagen, Germantown, MD, United States, #205211) with random primers according to manufacturer’s protocol. Resulting cDNA was preamplified in 20 μl reactions using PowerUp SYBR Green Master Mix (Applied Biosystems, Waltham, MA, United States, #A25742), with 40 nM of each PCR primer and 4 μl of template cDNA. The following thermal profile was used: Initial denaturation: 95°C, 3 min; Cycle (20 cycles total): Denaturation, 95°C, 20 sec; Amplification, 57°C, 3 min; Extension: 72°C, 1 min; Final extension: 72°C, 10 min. Primers were checked for specificity and correct product length by regular RT-PCR followed by agarose gel electrophoresis. Semi quantitative RT-PCR was performed using the PowerUp SYBR Green Master Mix (Applied Biosystems, Waltham, MA, United States) and preamplified cDNA as a template with the following thermal profile: 50°C for 2 min; 95°C for 2 min; 40 cycles of 95°C for 15 s, 57°C for 20 s, 72°C for 1 min; Final step: 95°C for 15 s, 57°C for 1 min, and 95°C for 15 s. All samples were run in duplicate in the QuantStudio 6 Flex Real Time PCR system (Applied Biosystems, Waltham, MA, United States). Relative gene expression was calculated with the ΔΔCt method after normalization to glyceraldehyde-3-phosphate de-hydrogenase (*GAPDH)* as reference gene.

### Next generation ribonucleic acid sequencing

Gene expression was assessed on RNA obtained from O4^+^ FACS sorted cells. RNA integrity was measured with the Agilent 2100 BioAnalyzer by next generation RNA sequencing (RNA-seq), and only RNA with RNA integrity number (RIN) above 7 was used for sequencing. Library preparation and sequencing was conducted by experienced personnel at the John P. Hussman Institute for Human Genomics (Miller School of Medicine, University of Miami) using an Illumina NovaSeq sequencing system. Paired-end, 125 base pair reads were generated. Reads were trimmed using Trim Galore v0.6.4 and aligned to hg39 human reference genome (UCSC Genome Browser) using default parameters. STAR was used to map the number of read pairs aligned to each gene. Differential gene expression analysis was conducted using EdgeR to generate *p*-values and calculate normalized expression measures of each gene in Fragments Per Kilobase per million Mapped reads (FPKM). Only genes with a log_2_CPM > 1 and log_2_| FC| ≥ 0.585 were included in heatmaps, volcano plots, and DAVID Gene Ontology (GO) Term analysis.

### Statistical analysis

Statistical analyses were performed using GraphPad Prism 9 software. Most data sets were analyzed with Student’s *t*-test with Welch’s correction, after testing for normality with the Shapiro–Wilk test. Sholl analysis data were analyzed by 2-way ANOVA with Bonferroni test for multiple comparisons. Data are presented as mean ± SEM, with *p*-values equal or lower than 0.05 considered as statistically significant.

## Results

### Control and primary progressive multiple sclerosis human induced pluripotent stem cell lines efficiently differentiate into O4 and myelin basic protein expressing oligodendrocytes

**FIGURE 1:**
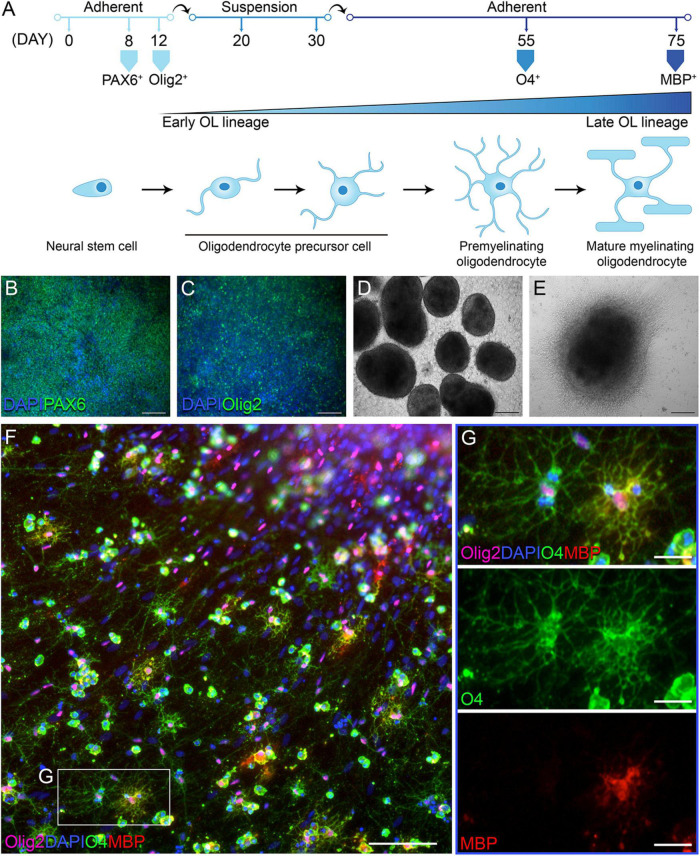
Differentiation of human induced pluripotent stem cells (hiPSCs) into oligodendrocytes (OLs). **(A)** Schematic of oligodendrocyte (OL) differentiation protocol and timeline, with cell markers identifying their state of maturity over time. **(B)** Day 8 of differentiation: neural progenitor state with cells expressing PAX6; scale bar: 100 μm. **(C)** Day 12 of differentiation: cells commit to the OL lineage and express Olig2; scale bar: 100 μm. **(D)** Day 30 of differentiation: cells form aggregates in suspension; scale bar: 200 μm. **(E)** Day 55 of differentiation: replated aggregates grow adherent and cells migrate outward; scale bar: 200 μm. **(F,G)** Day 76 of differentiation: immunofluorescent labeling with OL lineage markers Olig2, O4, and myelin basic protein (MBP); nuclei are stained with DAPI; scale bars: 100 μm **(F)** and 25 μm **(G)**.

In order to address whether OLs in individuals with PPMS are intrinsically different from OLs in unaffected individuals, we differentiated PPMS and control hiPSCs into OLs, adopting and optimizing the so-called “fast protocol” established by Douvaras and Fossati (2015). Following a 75 day-long differentiation protocol, all hiPSC lines were successfully and reproducibly differentiated into Olig2^+^ OLs expressing O4 and myelin basic protein (MBP) to varying degrees (Figure 1A). Cells were checked for appropriate expression markers throughout the process by immunostaining. As expected, the neural stem cell marker PAX6 was the first to be expressed around day 8 as the differentiation begun (Figure 1B). This was followed by Olig2 at day 12, indicating the cells had committed to the OL lineage (Figure 1C). At day 30, uniform round aggregates grown in suspension and with a diameter between 300 and 800 μm were hand selected (Figure 1D), replated, and cultured in mitogen-free medium for the remainder of the time. Expression of the OL marker O4 was observed by day 55 (Figure 1E), and combined O4 and MBP expression was detected in a small subset of cells by day 75 (Figures 1F,G). The percentages of differentiated cells expressing O4 and O4/MBP combined is in line with published works that adopted this differentiation paradigm (Douvaras and Fossati, 2015; Morales Pantoja et al., 2020).

### O4^+^ cells are reduced in number and are less ramified in primary progressive multiple sclerosis compared to control cell lines

**FIGURE 2:**
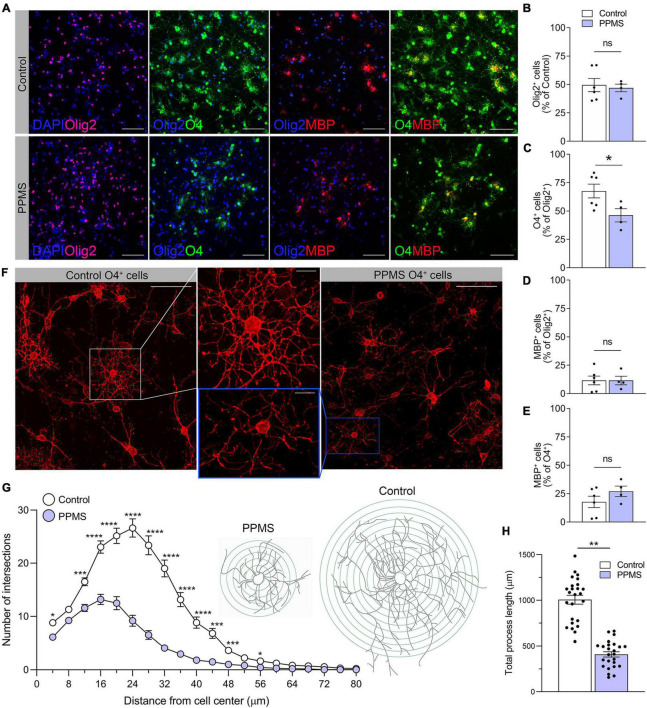
Quantification and morphological analysis of cell populations differentiated from control and primary progressive multiple sclerosis (PPMS) human induced pluripotent stem cells (hiPSCs). **(A)** Representative images of Olig2^ +^, O4^+^, and myelin basic protein (MBP^+^) cells in differentiated hiPSCs; scale bar: 100 μm. **(B)** Quantification of Olig2^+^ cells in the total cell population by HCA. **(C)** Quantification of O4^+^ cells within the Olig2^+^ population by high content analysis (HCA); *p ≤ 0.05, Student’s *t*-test. **(D)** Quantification of MBP^+^ cells within the Olig2^+^ population by HCA; **(E)** Quantification of MBP^+^ cells within the O4^+^ population by HCA. **(F)** Representative confocal images of O4^+^ Control and PPMS cells denoting morphological differences; scale bars: 25 and 10 μm (high magnification insert). **(G)** Sholl analysis of ramification and complexity of O4^+^ Control and PPMS oligodendrocytes, where the number in process intersections was quantified at increasing distance (4 mμ increments) from the cell center, *n* = 25/group, *p ≤ 0.05, ***p ≤ 0.001, ****p ≤ 0.0001, 2-way ANOVA, Bonferroni test. **(H)** Quantification of total process length/cell of O4^+^ Control and primary progressive multiple sclerosis (PPMS) OLs; *n* = 25/group, **p ≤ 0.001, Student’s *t*-test.

At the end of the 75 days differentiation period, we saw no difference in the number of Olig2^+^ OLs between control and PPMS lines (Figures 2A,B). However, within the Olig2^+^ population, PPMS cell lines displayed a significantly lower percentage of O4^+^ cells compared to controls (control 67.6 ± 6.1 vs. PPMS 46.2 ± 5.8, *p* = 0.0357, *t*-test) (Figures 2A,C). Notably, we observed no differences in the percentage of MBP^+^ cells within the Olig2^+^ and O4^+^ populations between PPMS and control lines (Figures 2A,D,E). This suggests that PPMS cells have reduced capacity to differentiate into O4^+^ OLs, but, once they reach this state, their potential to progress toward terminally differentiated cells capable of producing myelin components is maintained. It should be noted that, with the “fast” differentiation protocol we employed (Douvaras and Fossati, 2015), the number of cells reaching full maturation into MBP expressing cells was expectedly low, and we cannot exclude that, if the differentiation was prolonged further to enrich for the MBP^+^ population, we may have observed differences between conditions. Interestingly, O4^+^ PPMS derived OLs showed clear morphological differences compared to control cells, in that they appeared dystrophic with lower cell branching and complexity (Figure 2F). This was confirmed by Sholl analysis, where a marked and significant reduction in the number of process intersections, or nodes, was measured in PPMS compared to control O4^+^ OLs (Figure 2G). Additionally, a significant reduction in the total process length was measured in PPMS cells (Figure 2H).

### O4^+^ oligodendrocytes from primary progressive multiple sclerosis cell lines show transcriptional differences compared to control cell lines

**FIGURE 3:**
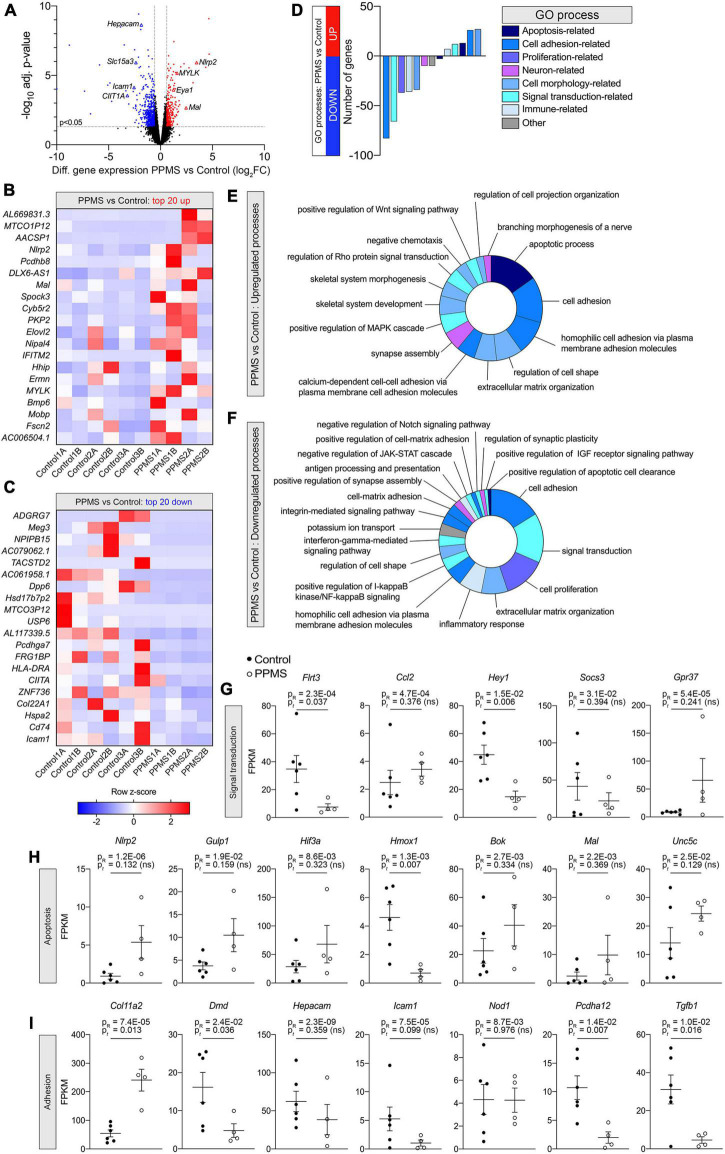
Differential gene expression analysis of O4^+^ cells differentiated from human induced pluripotent stem cells (hiPSCs) of primary progressive multiple sclerosis (PPMS) and control individuals. **(A)** Volcano plot of differentially expressed genes in PPMS vs. Control O4^+^ cells (blue: downregulated; red: upregulated). **(B,C)** Heatmaps of the top 20 upregulated **(B)** and downregulated **(C)** genes in PPMS vs. Controls cells. **(D)** DAVID Gene Ontology (GO) process analysis in PPMS vs. Controls cells. **(E,F)** Doughnut charts with the percentage of upregulated **(E)** and downregulated **(F)** genes in each process/pathway. **(G,I)** Gene expression profiles of select genes associated with signal transduction **(G)**, apoptosis **(H)**, and adhesion **(I)** processes. Gene expression is shown as fragments per kilobase per million mapped reads (FPKM). Average ± SEM of 4–6 samples/condition; *P*-values calculated with R and unpaired *t*-test with Welch’s correction are shown.

To better understand whether intrinsic abnormalities distinguished PPMS OLs from control OLs and these could contribute to their reduced differentiation into O4^+^ cells, we performed RNA-seq of pure O4^+^ OLs isolated by FACS after 76 days of differentiation. Each hiPSC line was independently differentiated two separate times, and samples for sequencing were obtained from both differentiations. Bioinformatics analysis with EdgeR revealed that 795 genes were significantly differentially expressed between the two groups, 249 upregulated (31.3%) and 546 downregulated (68.7%) in PPMS lines compared to controls (Figure 3A). Among the top 20 upregulated and downregulated genes, many were associated with apoptosis, cell adhesion, and immunomodulation (Figures 3B,C). This was further highlighted by DAVID GO term analysis showing that upregulated genes were enriched in apoptotic, cell adhesion, and morphology related terms (Figures 3D,E), while downregulated genes were noticeably associated with cell adhesion and signal transduction (Figures 3D,F). Downregulated signal transduction pathways included JAK-STAT, IGF-R, and IFN signaling (Figure 3F). Notably, genes associated with positive regulation of IGF-R signaling were downregulated in PPMS cells, and this could have negative implications for OL growth and differentiation, given the role of IGF-R signaling in these processes in the CNS (Dyer et al., 2016). Interestingly, *inflammatory response* process and positive regulation of NF-κB signaling were also downregulated, signifying a potential anti-inflammatory effect and, in general, a broad dysregulation of the immunomodulatory function of PPMS cells.

Looking deeper into the differentially regulated genes in each category, various genes associated with immunomodulatory and inflammatory function were identified. These included the chemokine *Ccl2*, upregulated in PPMS (Figure 3G), as well as the suppressor of inflammatory signaling *Socs3* and the antioxidant *Hmox1*, which were instead downregulated in PPMS cells (Figures 3G,H). Notably, the inflammasome component *Nlrp2* was upregulated in PPMS cells (Figure 3H), corresponding to the fourth top significantly upregulated gene in our screen (Figure 3B), despite sample variability. This is especially significant since, like other inflammasomes, NLRP2 is an innate immune sensor that participates in both apoptotic and inflammatory processes (Govindarajan et al., 2020) and its presence has never been reported in OLs. Adhesion-related genes were also differentially expressed, and most of them downregulated. These included, among others, *Hepacam*, *Icam1*, *Pcdhg3*, and *Dmd*, the gene encoding for dystrophyn, all molecules important for cell-cell contact and interaction, as well as motility (Figure 3I). Genes related to OL lineage specification and myelination were not notably different between PPMS and control O4^+^ OLs (Table 3).

**TABLE 3:** Oligodendrocyte-specific and myelin related genes expressed in primary progressive multiple sclerosis (PPMS) and Control O4^+^ cells.

Gene ID	Gene name	Differential gene expression PPMS vs. Control (logFC)	*P*-value (EdgeR)
ENSG00000105695	MAG	0.9126	0.0063*
ENSG00000123560	PLP1	0.7165	0.1537
ENSG00000204655	MOG	0.5511	0.0024*
ENSG00000173786	CNP	0.4765	0.1320
ENSG00000197971	MBP	0.4718	0.2096
ENSG00000100146	SOX10	0.2105	0.4178
ENSG00000125820	NKX2-2	0.2074	0.1271
ENSG00000054983	GALC	0.1203	0.5417
ENSG00000205927	OLIG2	−0.1246	0.4939
ENSG00000184221	OLIG1	−0.1924	0.2818
ENSG00000177468	OLIG3	−0.7587	0.6629

*Statistically significant.

**FIGURE 4:**
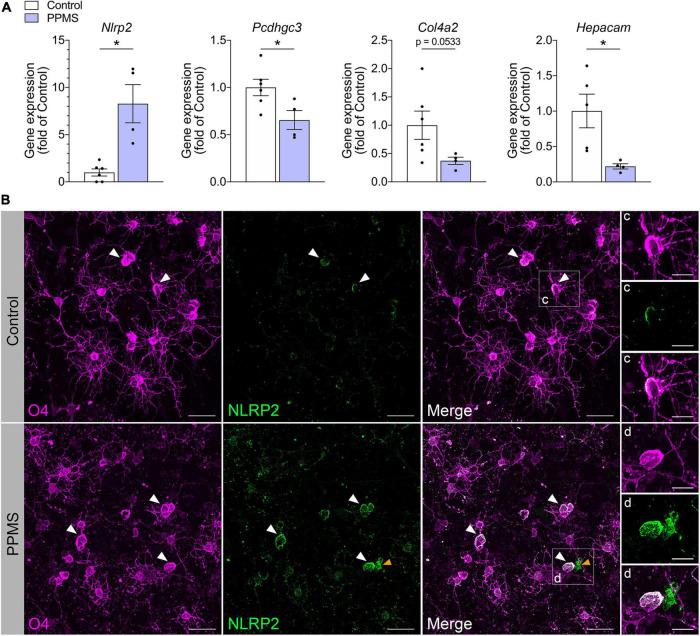
Validation of RNA-seq findings in differentiated cells at day 76. **(A)** qPCR analysis of select genes identified to be differentially expressed in primary progressive multiple sclerosis (PPMS) vs. Control O4^+^ cells in our RNA-seq screen. Relative gene expression was calculated after normalization to GAPDH with the 2^– ΔΔ^ Ct method; Average ± SEM of 4–6 samples/condition, *p ≤ 0.05, unpaired *t*-test with Welch’s correction. **(B–D)** Immunofluorescent staining for O4 and NLRP2 in oligodendrocytes (OLs) differentiated from control and PPMS induced pluripotent stem cell (iPSC) lines (day 76); white arrowheads: O4^+^NLRP2^+^ OLs; yellow arrowheads: O4^–^NLRP2^+^ astrocyte; Scale bars: 25 μm **(B)**, 10 μm **(C,D)**.

We validated our findings by quantifying with qPCR the expression of select differentially expressed genes identified in our screen (Figure 4A). Consistent with the RNA-seq data, *Pcdhgc3*, a protocadherin gene important for maintenance of cell-cell connections in the brain, *Col4a2*, and *Hepacam* were significantly downregulated in PPMS O4^+^ cells, while *Nlrp2* was upregulated.

Together, our data suggest that PPMS O4^+^ cells are phenotypically different from control cells, displaying an increased immunomodulatory profile and, in parallel, lacking the full repertoire of molecules necessary for interacting with the environment and neighboring cells.

### NLRP2 protein is upregulated in primary progressive multiple sclerosis O4^+^ cells and in oligodendrocytes from post-mortem primary progressive multiple sclerosis tissues

Among the various immunomodulatory genes differentially regulated in PPMS vs. control O4^+^ cells, *Nlrp2* stood out due to two reasons: first, its dual role in inflammatory and apoptotic processes, and second, the fact that its expression was previously only reported in astrocytes within the CNS, never in the OL lineage. To further investigate whether NLRP2 could be implicated in OL dysfunction in PPMS, we evaluated by immunofluorescent labeling NLRP2 protein expression and localization in hiPSC-derived OLs after 75 days in culture (Figure 4B). Control cells were shown to express NLRP2 sparsely and at low levels (Figures 4B,C), while PPMS cells showed increased and widespread expression compared to controls (Figure 4D). Additionally, we observed a few NLRP2^+^O4^–^ cells in our cultures, likely corresponding to NLRP2^+^ astrocytes based on previous reports (Minkiewicz et al., 2013), as well as the presence of astrocytes in our cultures. It must be underscored that astroglial expression of *Nlrp2* did not influence the *Nlrp2* gene expression profile in our RNA-seq experiment, which was conducted on purified FACS-sorted O4^+^ cells.

**FIGURE 5:**
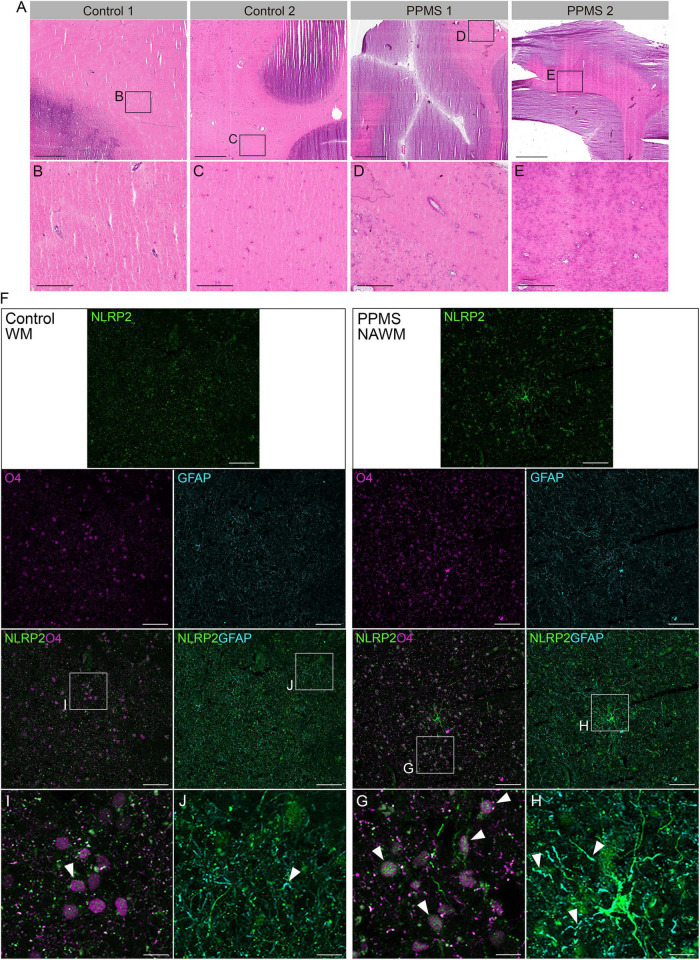
Histological analysis of control and primary progressive multiple sclerosis (PPMS) post-mortem human brain tissue. **(A–E)** Hematoxylin and eosin (H&E) staining of normal white matter (NWM) in controls **(B,C)** and normal appearing white matter (NAWM) in PPMs tissues **(D,E)**, showing absence of immune cell infiltrates; Scale bars: panels within **(A)** are equal to 500 μm, except **(H,I)** which equal to 100 μm. **(F–H)** Immunofluorescent staining for NLRP2 in combination with O4 oligodendrocytes (OLs) **(G,I)** and GFAP (astrocytes) **(H,J)** in normal white matter (NWM) in control and normal appearing white matter (NAWM) in PPMS tissues; Scale bars: panels within **(F)** equal to 50 μm, except **(H,I)** which equal to 10 μm.

To determine whether our *in vitro* data were consistent with NLRP2 expression *in vivo* and not a mere artifact of the culturing process, we analyzed the NAWM of post-mortem PPMS brain tissues and compared it to the subcortical NWM of control brains. The NAWM was specifically selected to ensure no immune infiltrates were present that could alter OL function, thus increasing the chances that any changes observed could be ascribed to intrinsic alterations. The absence of immune cell infiltrates was confirmed by H&E staining (Figures 5A–E). Confocal imaging of the tissues probed for O4, NLRP2, and GFAP showed clear expression of NLRP2 in O4^+^ OLs in PPMS samples, while NLRP2 was only minimally expressed in sparse O4^+^ cells in control tissues (Figures 5F,G,I). Furthermore, NLRP2 labeling was observed in astrocytes, as previously reported (Minkiewicz et al., 2013), also with higher expression in PPMS tissue (Figures 5H,J).

Our findings demonstrate, for the first time, not only the presence but the upregulation of the immunomodulatory protein NLRP2 in mature OLs of PPMS individuals, possibly implicating NLRP2 in intrinsic OL dysfunction in PPMS.

## Discussion

The molecular underpinnings of neurodegeneration that drive disease progression in MS and distinguish progressive MS forms, particularly PPMS, from the relapsing phenotype are still unknown. This is a crucial gap in knowledge that has hindered not only the development of effective therapies, but also the identification of biomarkers for the prompt diagnosis of progressive forms prior to irreversible CNS damage (Reich et al., 2018).

To contribute to a better understanding of PPMS pathology and to determine whether intrinsic dysfunction in OLs, the direct target of damage in MS, may contribute to PPMS pathogenesis, we took an *in vitro* approach and profiled the transcriptome of O4^+^ OLs differentiated from hiPSCs obtained from PPMS individuals and compared it to that of healthy controls in the absence of any exogenous inflammatory stimulation (e.g., cytokines). However, we can’t exclude the possibility that OLs may receive some inflammatory signals from other cells present in the culture, specifically astrocytes. Indeed, some astrocytes are generated through our differentiation protocol and what they produce may indirectly affect OL function. Nevertheless, we showed that PPMS-derived hiPSCs showed a reduced capacity to differentiate into O4^+^ OLs, but, once they reached this state, their potential to progress toward terminally differentiated cells capable of producing myelin components was maintained. This was shown by the fact that the number of MBP^+^ cells within the O4^+^ population did not differ between control and PPMS lines. Additionally, PPMS-derived O4^+^ cells displayed clear morphological abnormalities, with reduced process branching and length. We may speculate that this dystrophic phenotype could be associated with altered functionality, for example a diminished capacity to reach and wrap axons, and further experiments are warranted to address this possibility.

As we mapped the basal transcriptional signature of differentiated O4^+^ OLs, we found close to 800 differentially expressed genes between groups, the vast majority of which (about 70%) was downregulated in PPMS cells. Of the cellular processes most affected as a result of this reduced gene expression, many were related to cell adhesion, extracellular matrix (ECM) organization and integrin signaling. Because these are all important processes for myelination, this suggests that PPMS OLs may be impaired in their ability to make contact with axons and properly myelinate. Indeed, since activated integrins on the cell surface of OLs bind to the ECM and promote cellular adhesion prior to axon wrapping, it is likely that the combined dysregulation of multiple genes in these processes will have a major impact on the normal myelination capacity of OLs (Su et al., 2021). Notably, *Hepacam*, the gene that encodes for the adhesion molecule GlialCAM, was downregulated in PPMS OLs. Given that GlialCAM in the CNS is mainly found in OLs where it is upregulated during postnatal brain development concomitantly with MBP, it has been suggested that GlialCAM is important for myelination (Favre-Kontula et al., 2008). Thus, its downregulation in PPMS OLs further supports the idea of intrinsically impaired myelination capacity. It is also worth noting that higher titers of anti-GlialCAM antibodies are found in MS individuals and have been indicated as likely contributors to MS pathogenesis due to the molecular mimicry between GlialCAM and EBNA1 produced by Epstein-Barr virus (Lanz et al., 2022). Interestingly, our data show a reduced expression of the *Hepacam* gene in PPMS OLs, thus it will be important to assess whether the corresponding GlialCAM protein follows a similar pattern, particularly in PPMS post-mortem tissue. If this is the case, it is plausible that is the astroglia-derived GlialCAM that may contribute to pathology in PPMS.

Our transcriptomics analysis also uncovered an upregulation of genes associated with Wnt signaling in PPMS OLs, leading to aberrant positive regulation of the pathway. This is significant since OL differentiation requires suppression of Wnt signaling both in development and disease (Ye et al., 2009; Langseth et al., 2010; Niu et al., 2021; Gao et al., 2022), indicating that PPMS OLs may have an impaired maturation capacity, which is a prerequisite for repair in MS. Furthermore, aberrant Wnt tone in OPCs mediates their dysfunctional vascular interaction and compromises tight junction integrity, thus altering BBB function (Niu et al., 2019). This could be another mechanism by which dysfunctional OLs in PPMS contribute to pathology.

Consistent with recent reports that OL lineage cells acquire inflammatory and immunomodulatory function when exposed to stress/inflammatory conditions (Kirby et al., 2019; Fernandez-Castaneda et al., 2020; Harrington et al., 2020; Madsen et al., 2020; Desu et al., 2021; Kirby and Castelo-Branco, 2021), our screen showed dysregulated expression of genes associated with immune activation in PPMS OLs, including *HLA-B*, *HLA-DRA*, and *CIITA*, all important for antigen presentation. Notably, genome wide association studies have shown that mutations in a variety of *HLA* alleles are associated with increased MS risk (International Multiple Sclerosis Genetics Consortium Hafler et al., 2007). Additionally, *Socs3*, a suppressor of inflammation that negatively regulates cytokine signaling (Carow and Rottenberg, 2014), was downregulated in PPMS OLs indicating the potential failure of a feed-back mechanism designed to keep inflammatory activation in check. Collectively, these transcriptional changes, which are observed in the absence of any stimulation, support the idea that PPMS OLs have intrinsic abnormalities that could render them more inflammatory and pathogenic, especially when challenged by toxic factors such as those found in the MS environment. This is also in line with findings from snRNA-seq studies in post-mortem MS tissues describing the existence OL populations that exhibit an enhanced immune-inflammatory phenotype (Falcao et al., 2018; Schirmer et al., 2019; Meijer et al., 2022).

Further demonstration of the enhanced inflammatory signature of PPMS OLs in basal conditions is their strong upregulation of the inflammasome gene *Nlrp2*, confirmed at the protein level in both PPMS OLs and OLs in the NAWM of post-mortem MS tissues. Inflammasomes, particularly NLRP3, have been implicated in MS pathophysiology and are considered promising therapeutic targets and biomarkers, especially for PPMS (Boziki et al., 2020; Govindarajan et al., 2020; Malhotra et al., 2020; Lunemann et al., 2021). To this end, in our own work in the experimental autoimmune encephalomyelitis (EAE) model of MS, we showed that pan-inflammasome inhibition with a novel monoclonal antibody against the inflammasome adaptor protein ASC improved the clinical outcome (Desu et al., 2020). As far as the less investigated NLRP2 member of the inflammasome family, our study is the first to report its expression in OL lineage cells. Indeed, to date NLRP2 expression has only been described in astrocytes (Minkiewicz et al., 2013). Here, we identified NLRP2 expression and upregulation not only in PPMS hiPSC-derived OLs, but also in OLs within the NAWM of PPMS post-mortem tissues. Thus, we speculate that NLRP2 upregulation may be associated with PPMS-specific OL dysfunction and possibly contribute to PPMS pathology. We realize that measures of inflammasome assembly and activation (e.g., production of IL1β and IL18, cleaved caspase-1 presence) are necessary to validate this hypothesis and they are being conducted in ongoing studies in the lab. Additionally, we will explore whether NLRP2 detection in the cerebrospinal fluid of individuals with PPMS may serve as a biomarker for disease phenotyping and/or progression.

Ultimately, the key finding of our study is that PPMS-derived OLs have an intrinsically abnormal transcriptional and morphological signature that may drive cell dysfunction and potentially contribute to PPMS pathogenesis and/or progression. However, this may not be the case in all MS forms. Indeed, a recent study reported that, with respect to myelination-associated functions and proteomic composition, OLs differentiated from iPSCs of individuals with RRMS are indistinguishable from OLs derived from control iPSCs (Mozafari et al., 2020). However, after exposure to extrinsic factors released by activated peripheral blood mononuclear cells (PBMCs), their differentiation capacity is impaired, suggesting this could contribute to the remyelination failure observed in MS. Since our study demonstrates that OLs differentiated from iPSCs of individuals with PPMS are intrinsically different from control OLs in the absence of stimulation, this may help explain the different pathological characteristics, clinical phenotypes and outcomes of RRMS vs. PPMS, reinforcing the concept that PPMS may be associated with specific and unique pathogenic mechanisms. It is also possible that intrinsic OL dysfunction may be a common feature of all progressive MS forms. Indeed, OPCs derived from iPSCs of individuals with SPMS have also been shown to display an altered gene expression profile compared to controls OPCs as well as a different secretome (Lopez-Caraballo et al., 2020). Similar to our data, SPMS-derived OPCs showed dysregulation of genes implicated in cell adhesion, communication and differentiation. Additionally, neural progenitor cells (NPCs) differentiated from iPSCs of PPMS individuals fail to differentiate into OPCs due to cellular senescence driven by the intrinsic upregulation of high-mobility group box-1 (HMGB1) (Nicaise et al., 2019). Reversal of this senescent phenotype by rapamycin exposure restores OPC maturation, suggesting that senescence could be an active process in progressive MS that can explain, at least in part, the limited remyelination and repair capabilities observed in these phenotypes.

In summary, our work demonstrated that PPMS-derived OLs have an intrinsically abnormal transcriptional profile that could impact their ability to properly interact with the CNS environment, particularly neurons, thus leading to pathology. We provided one more example that hiPSCs are indeed a useful tool to learn about the phenotype, function and potential pathological alterations of brain-specific cells in MS, not only neural cells, but other CNS-restricted types as well, e.g., brain microvascular endothelial cells (Nishihara et al., 2022), that would otherwise be impossible to study *in vivo*. Importantly, we generated a toolbox of genes for which further exploration is warranted in order to discern the mechanisms that underlie PPMS pathology and differentiate it from other MS forms. Our expectation is that selective targeting of such mechanisms will eventually lead to viable and much needed therapeutic options for PPMS.

## Data availability statement

All RNAseq datasets generated for this study are publicly available in the NIH Gene Expression Omnibus (GEO) repository: https://www.ncbi.nlm.nih.gov/geo/, accession number GSE211739.

## Ethics statement

Brain tissues were obtained from the United Kingdom Multiple Sclerosis Tissue Bank at Imperial College London *via* a United Kingdom prospective donor scheme with full pre-mortem consent and ethical approval (United Kingdom Ethics Committee 08/MRE09/31). The patients/participants provided their written informed consent to participate in this study.

## Author contributions

MP designed and conducted experiments, analyzed data, and wrote the manuscript. HD analyzed RNA-seq data and aided in cell culture differentiation. MA performed Sholl analysis. AL performed confocal imaging. MS provided a control hiPSC cell line and contributed to the design and execution of cell differentiations. RB conceived the study, designed experiments, analyzed data, and wrote the manuscript. All authors reviewed and edited the manuscript.
